# Mass spectrometry-based proteomic analysis of middle-aged vs. aged *vastus lateralis* reveals increased levels of carbonic anhydrase isoform 3 in senescent human skeletal muscle

**DOI:** 10.3892/ijmm.2012.1056

**Published:** 2012-07-06

**Authors:** LISA STAUNTON, MARGIT ZWEYER, DIETER SWANDULLA, KAY OHLENDIECK

**Affiliations:** 1 Department of Biology, National University of Ireland, Maynooth, Kildare, Ireland;; 2 Department of Physiology II, University of Bonn, D-53115 Bonn, Germany

**Keywords:** aging, carbonic anhydrase isoform 3, difference in-gel electrophoresis, sarcopenia, *vastus lateralis*

## Abstract

The age-related loss of skeletal muscle mass and associated progressive decline in contractile strength is a serious pathophysiological issue in the elderly. In order to investigate global changes in the skeletal muscle proteome after the fifth decade of life, this study analysed total extracts from human *vastus lateralis* muscle by fluorescence difference in-gel electrophoresis. Tissue specimens were derived from middle-aged (47–62 years) vs. aged (76–82 years) individuals and potential changes in the protein expression profiles were compared between these two age groups by a comprehensive gel electrophoresis-based survey. Age-dependent alterations in the concentration of 19 protein spots were revealed and mass spectrometry identified these components as being involved in the excitation-contraction-relaxation cycle, muscle metabolism, ion handling and the cellular stress response. This indicates a generally perturbed protein expression pattern in senescent human muscle. Increased levels of mitochondrial enzymes and isoform switching of the key contractile protein, actin, support the idea of glycolytic-to-oxidative and fast-to-slow transition processes during muscle aging. Importantly, the carbonic anhydrase (CA)3 isoform displayed an increased abundance during muscle aging, which was independently verified by immunoblotting of differently aged human skeletal muscle samples. Since the CA3 isoform is relatively muscle-specific and exhibits a fibre type-specific expression pattern, this enzyme may represent an interesting new biomarker of sarcopenia. Increased levels of CA are indicative of an increased demand of CO_2_-removal in senescent muscle, and also suggest age-related fibre type shifting to slower-contracting muscles during human aging.

## Introduction

Although natural aging of the body may be considered a fundamental biological process (1), it is not clear which molecular and evolutionary genetic mechanisms trigger the progressive decline in physiological functions and decrease the rate of reproduction (2). Aging is most likely not due to active gene programming (3), but more likely based on evolved limitations in somatic maintenance (4). In humans, one of the most striking features of aging is a gradual reduction in skeletal muscle mass and a concomitant decline in contractile strength (5). This age-related progressive loss of muscle mass and function has been termed sarcopenia and is believed to be due to a multifactorial etiology (6–8). As reviewed by Berger and Doherty (9), epidemiological studies of age-related skeletal muscle wasting indicate that nearly half the population over 75 years of age is suffering from some form of sarcopenia leading in severe cases to loss of independence. Although the findings from a large number of longitudinal and cross-sectional studies of skeletal muscle aging (10–19) do not concur on the exact rate and extent of contractile tissue loss (20), these investigations agree that human aging is associated with a general impairment of structural and functional elements of the musculoskeletal system (21). Since individual skeletal muscles are differentially affected during the natural aging process (22), one should be careful about extrapolating age-related changes in a particular muscle group to the aging process of the entire neuromuscular system.

Muscle wasting occurs in all aged individuals to a varying degree (23), but this degenerative process may be accelerated by a variety of exacerbating factors, such as lack of physical activity, improper nutritional intake, extensive pharmacotherapy and/or chronic illness (24). For example, extended periods of bed rest in elderly patients are clearly related to inactivity-induced insulin resistance and further complicate the sarcopenic syndrome (25). Advancing age is associated with the loss of spinal motor neurons due to apoptosis (26), an impaired capacity for axonal re-innervation of deinnervated muscle fibers (27) and age-induced low-grade inflammation (28). Histological hallmarks of sarcopenia are a reduction in fibre numbers, a decline in fibre size and potential loss of entire motor units, whereby preferential atrophy of type II fibres has been established as a consequence of aging (21,27,29). The diagnosis of sarcopenia is based on the combined presence of a low muscle mass and a low gait speed (30–32). The clinical cut-off point for sarcopenia is considered to be a percentage of muscle mass two standard deviations below the mean measured in young adults of the same gender and ethnic background, as well as a walking speed below 0.8 m/sec in the 4 m walking test (31).

Since a reduction in skeletal muscle mass is pronounced in aged lower limb muscle groups, the human *vastus lateralis* muscle has been the center of attention in numerous studies on old age (33). Between the ages of 20 and 80 years, the cross-sectional area of the *vastus lateralis* muscle may be reduced by up to 40% (34,35). Epidemiological studies have indicated accelerated muscle wasting after the fifth decade with an approximately 2% reduction in muscle mass per year (9). We carried out a comparative survey of total extracts from middle-aged vs. aged *vastus lateralis* muscle tissue. Fluorescence difference in-gel electrophoresis (DIGE) in combination with mass spectrometry (MS) was employed to study human muscle aging, since this advanced method of modern protein biochemistry is capable of swiftly evaluating potential changes in large numbers of protein species (36). Over the last decade, MS-based proteomics has identified numerous novel protein factors involved in myogenesis, muscle differentiation, fibre transitions and various neuromuscular pathologies, as summarised in recent reviews (37–39). With respect to the natural aging process, proteomics technology has been applied to study altered protein expression levels associated with cellular changes in skeletal muscle tissues (40), whereby most studies have focused on animal models of aging, such as the senescent Wistar rat (41–51).

A previous proteomic survey of human muscle aging has investigated differences in protein expression between young adults (20–25 years) and aged (70–76 years) individuals (52). In order to build on these findings and to determine potential changes after the fifth decade of life, we used fluorescent tagging of the *vastus lateralis* muscle proteome from middle-aged (47–62 years) vs. aged (76–82 years) individuals and conducted a comprehensive gel electrophoresis-based survey of human skeletal muscle aging. Densitometric scanning revealed a differential expression pattern for 19 2-dimensional (2D) protein spots and the subsequent mass spectrometric analysis identified these muscle-associated proteins as being involved in contraction, relaxation, ion homeostasis, cellular stress response and fibre metabolism. Alterations in key muscle proteins from *vastus lateralis* muscle support the idea of generally perturbed protein expression patterns during human aging and agree with the notion that sarcopenia is associated with the fast-to-slow and glycolytic-to-oxidative transition processes (52–54). Of note, the muscle-specific carbonic anhydrase (CA)3 isoform (EC 4.2.1.1) (55), which has not been previously identified by proteomic surveys of human muscle aging (38,52), showed an increase in senescent skeletal muscle by both MS-based proteomics and independent immunoblot analysis. Elevated levels of CA3 may be indicative of an increased demand for efficient CO_2_-removal during fibre aging and/or age-related fibre type shifting to slower-contracting muscle populations (54). The establishment of a novel biomarker of muscle aging after the fifth decade of life may be exploitable for the future development of a more reliable assay to diagnose sarcopenia in old age.

## Materials and methods

### Materials

For the fluorescence 2D-DIGE analysis of aged human skeletal muscles, immobilised pH gradient (IPG) drystrips pH 3–11, CyDye DIGE Fluor minimal dyes Cy3 and Cy5, Coomassie Brilliant Blue, ampholytes, acetonitrile, cover fluid, and the 2D-Clean-Up kit were purchased from Amersham Biosciences/GE Healthcare (Little Chalfont, UK). Ultrapure protogel acrylamide stock solutions were from National Diagnostics (Atlanta, GA, USA). Sequencing grade-modified trypsin for peptide generation was obtained from Promega (Madison, WI, USA). LC-MS Formic acid and Chromasolv water were purchased from Fluka Chemical Corp. (Milwaukee, WI, USA) and spin filters were from Fisher Scientific (Loughborough, UK). Chemiluminescence substrate, protease inhibitors and nitrocellulose sheets were from Pierce and Warriner (Chester, UK), Roche Diagnostics (Manheim, Germany) and Millipore (Bedford, MA, USA), respectively. For immunoblotting, primary antibodies to the CA3 isoform were purchased from Abcam (Cambridge, UK) and secondary peroxidase-conjugated antibodies were from Chemicon International, Inc. (Temecula, CA, USA). Ultrapure lysine for quenching the DIGE labelling reaction, DNase I and all general reagents were otained from Sigma Chemical Co. (Dorset, UK).

### Human skeletal muscle biopsy samples

Since the neuromuscular system of the *vastus lateralis* muscle is intensively studied in human physiology and biochemistry, and frequently targeted during biopsy procedures for diagnostic purposes or routine biomedical investigations, age-related changes in this muscle were studied by MS-based proteomics. Two groupings of biopsy material representing healthy middle-aged vs. healthy aged *vastus lateralis* muscle were provided by the associated hospitals of the University of Bonn according to German ethics regulations (56). Middle-aged specimens derived from 47-, 55-, 59- and 62-year-old muscle tissue are marked in this study as samples MA1 to MA4. Aged specimens derived from 76-, 77-, 81- and 82-year-old muscle tissue are marked as samples OA1 to OA4. Following biopsy, fresh tissue specimens were immediately frozen in liquid nitrogen, transported on dry ice and then stored at −80°C prior to usage.

### Preparation of urea-soluble fraction of skeletal muscle proteome

Freshly thawed skeletal muscle biopsy material (100 mg) was ground into a fine powder in the presence of liquid nitrogen using a pestle and mortar. Pulverised human muscle specimens of different ages were resuspended in 1 ml of ice-cold buffer containing 7 M urea, 2 M thiourea, 65 mM CHAPS, 10 mM Trizma base, 1% ampholytes pH 3–11, and 100 mM dithiothreitol. In order to eliminate excessive viscosity of the protein extract due to large amounts of muscle-derived DNA and to minimise protein degradation due to the presence of muscle-associated proteases, the solution was supplemented with 2 *μ*l of DNase I (200 units; Sigma Chemical Co.) per 100 *μ*l buffer and a protease inhibitor cocktail (Roche Diagnostics) (57). The muscle tissue homogenate was gently mixed by vortexing and then placed on a bench top shaker for 2.5 h at room temperature to precipitate the total urea-soluble protein complement. Following centrifugation of the suspension at 20,000 x g in an Eppendorf 5417R centrifuge (Eppendorf, Hamburg, Germany) for 20 min, the protein-containing pellet was washed in 5 ml of ice-cold 100% acetone and thoroughly broken up by vortexing and sonication. Using 80% acetone, washing and centrifugation was repeated twice and the final protein precipitate was collected by centrifugation and resuspended in 1 ml of the above described buffer. Individual protein samples were incubated for 1 h at room temperature with careful vortexing every 10 min for 5 sec. Determination of protein concentration and removal of potentially interfering contaminants was performed using the Bradford assay system (58) and 2D-Clean-Up kit from Amersham Biosciences/GE Healthcare (59), respectively. Finally, protein pellets representing middle-aged and aged skeletal muscle protein fractions were resuspended in DIGE lysis buffer (7 M urea, 2 M thiourea, 30 mM Tris, 4% CHAPS, pH 8.5) and adjusted to a protein concentration of 1 mg/ml.

### Fluorescence DIGE analysis

The gel electrophoretic separation of fluorescently tagged muscle proteins was performed with a total amount of 100 *μ*g protein per 2D-DIGE gel. To keep potential impurities to a minimum, electrophoretic separation steps were performed under designated fume hoods. For each protein preparation derived from differently aged *vastus lateralis* muscle, a 50 *μ*g protein aliquote was fluorescently-labelled with 200 pmol DIGE fluor dyes for 30 min on ice and in the dark. Tissue extracts from eight different muscle samples representing 47–82 years of age were each labelled with Cy3 dye. In addition, pooled internal standards were prepared with Cy5 dye, as previously described in detail (60). Quenching of the fluorescent labelling reaction was carried out with 10 mM lysine for 10 min on ice. For separation in the first dimension, isoelectric focusing (IEF) was carried out with 24 cm strips in an Amersham IPGphor system using the following running conditions: 30 V for 2 h, 500 V for 2.5 h, 1,000 V for 1 h, 2,000 V for 1 h, 4000 V for 1 h, 6,000 V for 1 h, 800 V for 3 h, 500 V for 1.5 h and finally 8,000 V for 2.5 h. Gel strips were then equilibrated for 20 min in reducing buffer containing 100 mM dithiothreitol, followed by 10 min of alkylation in buffer containing 250 mM iodoacetamide. For separation in the second dimension, slab gel electrophoresis was carried out with an Amersham Ettan Dalt-twelve system, using 12.5% gels. Eight slab gels were run in parallel at 0.5 W/gel for 60 min and then 15 W/gel until the blue dye front had disappeared from the bottom of the gel. Cy3- and Cy5-labelled muscle-associated proteins were visualised with the assistance of an Amersham Typhoon Trio variable mode imager. Individual 2D-DIGE gels were warped to a single master gel prior to analysis. Potential changes in the protein expression profile of middle-aged vs. aged skeletal muscle samples were analysed using Progenesis SameSpots analysis software from Nonlinear Dynamics (Newcastle upon Tyne, UK), using the following parameters: n=4; t-test P<0.05; and a power value of >0.8. Muscle proteins with a significantly changed density were selected for tryptic digestion from Coomassie Brilliant Blue-stained preparative gels (61).

### Mass spectrometric identification of skeletal muscle proteins

The mass spectrometric identification of proteins of particular interest was carried out in a dedicated and air-conditioned proteomics suite with semi-clean analytical status to avoid potential contamination of samples. Protein spots were digested by standardised in-gel trypsination to generate distinct peptide populations (62). MS analysis was performed on a Model 6340 Ion Trap LC/MS apparatus from Agilent Technologies (Santa Clara, CA, USA). Spot excision, washing, destaining and treatment with protease were performed by previously optimised methods (57,60,61). Trypsin-generated peptide mixtures were dried through vacuum centrifugation and then resuspended in MS-grade distilled water and 0.1% (v/v) formic acid, spun down through spin filter and added to LC-MS viles for identification by ion trap LC-MS analysis. A nanoflow Aligent 1200 series system was employed for peptide separation. Analytical samples were loaded into the enrichment at a capillary flow rate set to 2 *μ*l/min with a mix of 0.1% (v/v) formic acid and 50% (v/v) acetonitrile and formic acid at a ratio of 19:1. The voltage was set to 1,700 V. Database searches were carried out with Mascot MS/MS Ion search (Matrix Science, London, UK). All searches used ‘*Homo sapiens*’ as a taxonomic category and the following parameters: i) two missed cleavages by trypsin, ii) mass tolerance of precursor ions ±2.5 Da and product ions ±0.7 Da, iii) carboxymethylated cysteins fixed modification, and iv) oxidation of methionine as variable modification. In addition, only hits with at least two matched distinct peptides and a Mascot score of at least 44 were considered significant proteomic findings.

### Verification of age-related changes in the expression of carbonic anhydrase by immunoblotting

In order to verify age-related changes in the abundance of CA, immunoblot analysis was carried out to determine the expression levels of the CA3 isoform in middle-aged vs. aged *vastus lateralis* muscle preparations. The electrophoretic transfer of muscle proteins to Immobilon NC-pure nitrocellulose membranes was carried out for 1 h at 100 V and 4°C with a Mini-Protean transfer system from Bio-Rad Laboratories (Hemel-Hempstead, UK). Transfer efficiency was routinely evaluated by reversible Ponceau S-Red staining of membrane sheets. Nitrocellulose membranes were blocked for 1 h in 5% (w/v) fat-free milk powder dissolved in phosphate-buffered saline [PBS; 50 mM sodium phosphate, 0.9% (w/v) NaCl, pH 7.4]. Blocked and washed membranes were incubated for 3 h at room temperature with appropriately diluted primary antibody to the CA3 isoform, followed by a gentle washing in blocking buffer and then incubated with an appropriate dilution of peroxidase-conjugated secondary antibody for 1 h at room temperature (57). Immuno-decorated membranes were washed again in blocking solution and a final rinse in PBS. Antibody-labelled protein bands were visualised with the SuperSignal-type enhanced chemiluminescence kit from Pierce and Warriner. Densitometric scanning of immunoblots was performed on a 300 S computing densitometer (Molecular Dynamics, Sunnyvale, CA, USA) with ImageJ (NIH, USA) and GraphPad Prism (GraphPad Software, Inc., San Diego, CA, USA) software.

## Results

**Figure 1:**
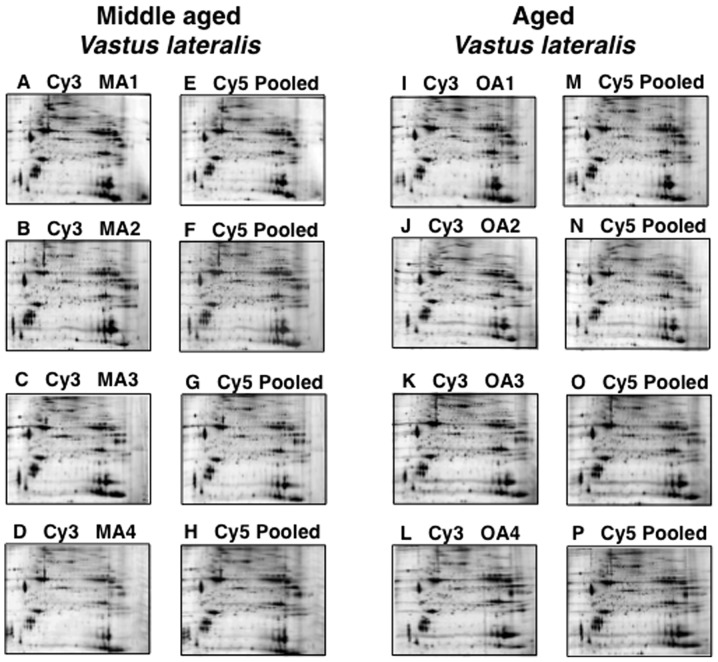
Two-dimensional (2D) fluorescent difference in-gel electrophoretic analysis of proteins during human skeletal muscle aging. Shown are fluorescent 2D gels of middle-aged (A–D) vs. aged (I–L) *vastus lateralis* muscle preparations, as well as pooled standards (E–H and M–P). Specimens MA1 to MA4 were derived from 47-, 55-, 59- and 62-year-old muscle tissue, and specimens OA1–OA4 were derived from 76-, 77-, 81- and 82-year-old muscle tissue, respectively. MA, middle-aged; OA, old-aged.

**Figure 2:**
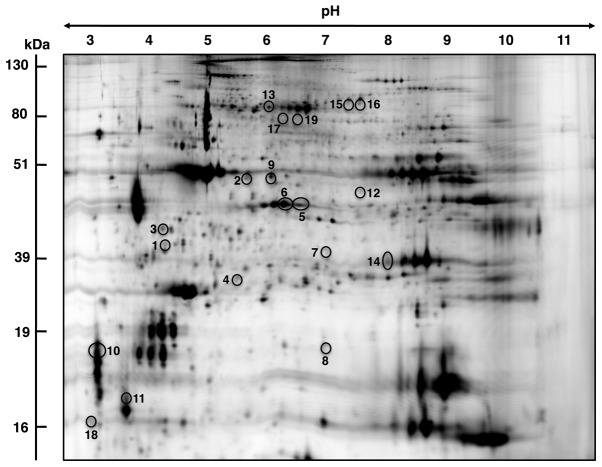
Proteomic identification of changed proteins during human skeletal muscle aging. Shown is a difference in-gel electrophoresis master gel of total protein extracts from *vastus lateralis* muscle. Skeletal muscle-associated proteins with a significantly different expression level are marked by circles and are numbered 1–19. Table I displays detailed listing of muscle proteins with a changed abundance in aged contractile tissue. The pH values of the first dimension gel system and molecular mass standards of the second dimension are indicated on the top and on the left of the panel, respectively.

**Figure 3:**
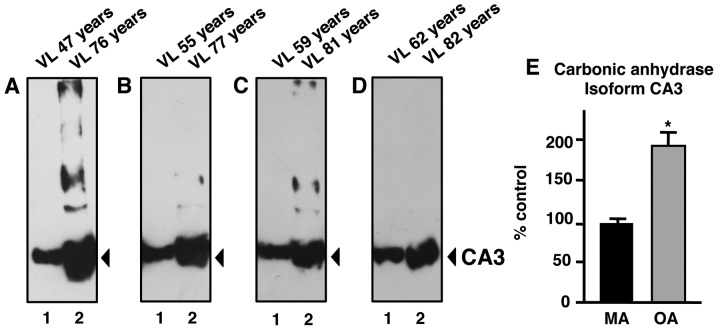
Immunoblot analysis of carbonic anhydrase CA3 in aged human skeletal muscle. Shown are representative immunoblots labelled with antibodies to the CA3 isoform. Lanes 1 and 2 represent middle-aged (MA) vs. old-aged (OA) *vastus lateralis* (VL) muscle specimens, respectively. Immunoblot panels show individual middle-aged samples vs. individual aged samples as follows: (A) 47 vs. 76 years, (B) 55 vs. 77 years, (C) 59 vs. 81 years, and (D) 62 vs. 82 years. (E) The comparative immunoblot analysis was statistically evaluated using an unpaired Student’s t-test (n=4; ^*^P<0.05).

In order to evaluate age-dependent alterations in the skeletal muscle proteome after the fifth decade of life, we applied the fluorescence 2D-DIGE technique and MS for studying *vastus lateralis* specimens from middle-aged vs. aged individuals. In our current study, the comparative gel electrophoresis-based proteomic study of crude muscle extracts focused on urea-soluble proteins, which is clearly reflected by the identification of mostly soluble and relatively abundant protein species. Shown is an overview of the analytical DIGE gels used in this proteomic study (Fig. 1), a DIGE master gel outlining protein spots with a significant age-related change in abundance (Fig. 2), a listing of changed proteins in senescent *vastus lateralis* muscle as determined by MS analysis (Table I) and immunoblotting of changed expression levels in a muscle-specific protein, the CA3 isoform (Fig. 3). In order to correlate MS-identified protein species listed in Table I with distinct protein spots of altered concentration in the DIGE master gel shown in Fig. 2, the numbering system of proteins in the table and corresponding gel is identical.

### Proteomic profiling of aging human vastus lateralis muscle

In this study, high-resolution fluorescence 2D gel electrophoresis, in combination with densitometric analysis using a Typhoon Trio variable imager and image analysis with progenesis 2D analysis software, resulted in the identification of 19 protein spots with a significant change in concentration levels in middle-aged vs. aged individuals. Shown are analytical DIGE gels of protein extracts from 47-, 55-, 59- and 62-year-old muscle tissue (Fig. 1A–D) and from 76-, 77-, 81-and 82-year-old muscle tissue (Fig. 1I–L), labelled with Cy3 dye. Pooled standards, labelled with Cy5 dye, are depicted in Fig. 1E–H and M–P.

### MS identification of skeletal muscle proteins with an age-related change in concentration

A DIGE master gel with electrophoretically separated human *vastus lateralis* muscle proteins is shown for a molecular mass range of approximately 10–150 kDa and a p*I* range of pH 3 to pH 11 (Fig. 2). Muscle-associated proteins with a potential age-dependent alteration in density ranged in molecular mass from apparent 16.7 kDa (calmodulin) to 97.7 kDa (glycogen phosphorylase) and covered a p*I* range from approximately 4.09 (calmodulin) to 8.96 (Acyl-CoA dehydrogenase). MS analysis identified 19 proteins species, which are listed in Table I outlining protein name, protein accession number, molecular mass, p*I*-value, Mascot score, percentage sequence coverage, number of matched peptide sequences, Anova score, fold change of individual proteins affected by muscle aging, and peptide sequences used to identify proteins. The majority of muscle-associated proteins identified by DIGE screening in combination with MS analysis were shown to be associated with the actomyosin apparatus, the cytoskeleton, metabolism, signalling and the cellular stress response. A reduced density was determined for 11 proteins and 8 proteins showed an increase in their abundance.

The *vastus lateralis* muscle protein with the highest fold decrease was shown in the muscle isoform of α-actin (spots 1, 2). Phosphoglucomutase (spot 19) was identified as the significantly increased enzyme in aged muscle tissue. In addition to muscle α-actin, muscle β-actin (spot 9), Annexin (spot 3), DJ-1 protein (spot 4), troponin subunits (spots 5, 6, 10), glycogen phosphorylase (spot 7), heat shock protein (Hsp) β-7 (spot 8), and myosin light chain (spot 11) were decreased in senescent human muscle. Besides phosphoglucomutase, increased muscle proteins were identified as Acyl-CoA dehydrogenase (spot 12), Hsp70 (spot 13), CA3 isoform (spot 14), muscle creatine kinase (spot 15), succinate dehydrogenase (spot 16), cardiac α-actin (spot 17) and calmodulin (spot 18).

In addition to the proteins listed in Table I, the DIGE method in combination with MS analysis identified two other proteins with a changed abundance in aged muscle; slow myosin light chain MLC3 (gi|33563264|; 22523 kDa, p*I* 5.03) and the voltage-dependent anion-selective channel VDAC2 (gi|6755965|; 32351 kDa, p*I* 7.44). The contractile protein, MLC3, was identified by six peptides (ALGQNPTQAEVLR, MMDFETFLPMLQHISK, NKDTGTYEDFVEGLR, EGNGTVMGAELR, HVLATLGER and LTEDEVEK) with a 35% sequence coverage and a Mascot score of 86, and the mitochondrial ion channel, VDAC2, was identified by three peptides (LTFDTTFSPNTGK, VNNSSLIGVGYTQTLRPGVK and LTLSALVDGKSFNAGGHK) with a 17% sequence coverage and a Mascot score of 64. Although their identification was based on a sufficient number of peptide sequences, the DIGE-based determination of their respective 2D spots displayed only minor fragments. Therefore, these proteins were not included in the listing of the main findings of this proteomic survey of human skeletal muscle aging.

### Immunoblot analysis of CA3 in aging human skeletal muscle

Although the DIGE technique represents one of the most powerful comparative methods in modern biochemistry and MS analysis is highly accurate in identifying distinct protein species, we were interested in independently verifying the differential expression of a muscle-specific protein identified in this proteomic study on old age. Since the CA3 isoform is a relatively muscle-specific and fibre type-specific enzyme, comparative immunoblotting was carried out to confirm a key finding of the proteomic data presented in this study. The increased immuno-decoration of CA in aged vs. middle-aged specimens from *vastus lateralis* muscle (Fig. 3A–D) is in agreement with the proteomic establishment of higher levels of this muscle enzyme in senescent contractile tissue (Fig. 2 and Table I). A comparison of 47- vs. 76-year-old, 55- vs. 77-year-old, 59- vs. 81-year-old and 62- vs. 82-year-old skeletal muscle specimens revealed a statistically significant increase in CA3 isoform expression in senescent skeletal muscle (Fig. 3E).

## Discussion

**Figure 4:**
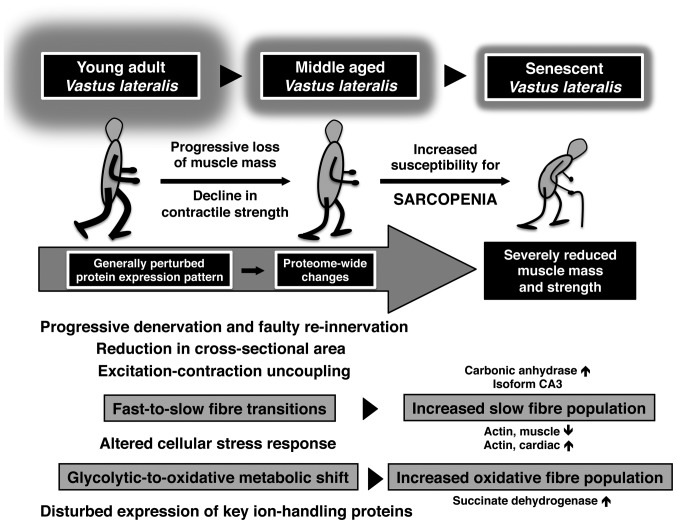
Diagrammatic overview of molecular and cellular changes during skeletal muscle aging. The flowchart summarises major age-related changes in contractile patterns and muscle metabolism as revealed by proteomic analyses of young adult vs. middle-aged vs. aged human *vastus lateralis* muscle.

The progressive decline in skeletal muscle mass and the weakening of contractile strength is a major pathophysiological feature of the aged neuromuscular system. Often the frailty syndrome and muscular dysfunction present personal care problems for elderly individuals, limiting their independence and requiring them to seek outside help, despite other medical ailments (63). This warrants large-scale genomic, proteomic and metabolomic surveys of aged skeletal muscle in order to establish the underlying mechanisms of sarcopenia in old age. Over the last few years, a considerable number of molecular investigations involved in the genetic basis of sarcopenia have identified a large collection of differentially expressed genes in aged muscle tissue (64). Based on these genetic findings, it is crucial to verify which of the identified age-dependent alterations in gene expression patterns translate into changed concentration levels and/or post-translational modifications in distinct protein products that markedly modify skeletal muscle functions. The MS-based proteomic survey of human aging presented in this study focused on the elucidation of potential alterations in the muscle protein complement after the fifth decade of life. Our fluorescent DIGE study of the *vastus lateralis* muscle compared middle-aged (47–62 years) vs. aged (76–82 years) individuals in order to supplement a set of previously determined proteomic data on the same skeletal muscle from young adults (20–25 years) and aged (70–76 years) individuals (52). Based on the proteome-wide changes revealed by these extensive gel electrophoresis-based surveys, the illustrative scheme in Fig. 4 summarises our knowledge of major molecular and cellular events during human skeletal muscle aging. Aged human *vastus lateralis* muscle tissue, which exhibits progressive denervation, faulty re-innervation and reduction in cross-sectional area (21,34), is characterised by excitation-contraction uncoupling, an altered cellular stress response, impaired ion homeostasis, fast-to-slow fibre transitions and glycolytic-to-oxidative metabolic shifts (52–54).

The comparative proteomic study of middle-aged vs. aged human muscle identified age-related changes in the expression of key proteins involved in the excitation-contraction-relaxation cycle, ion handling, the cellular stress response and muscle fibre bioenergetics. The apparent switch between the muscle isoform of actin with its cardiac counter-part (65) in aged *vastus lateralis* muscle supports the idea of a fast-to-slow transformation process in the actomyosin apparatus during skeletal muscle aging (54). However, the decrease in slow subunits of the regulatory element, troponin, and myosin light chain 2 (MLC2) does not follow this general trend of isoform switching in a slower-twitching aged muscle population. On the other hand, increased levels of the mitochondrial enzyme, succinate dehydrogenase, clearly support the metabolic concept of glycolytic-to-oxidative transitions during fibre aging (53). This proteomic finding agrees with the findings from numerous published studies on skeletal muscle aging in both rodent models of sarcopenia and senescent human muscle (46,50,52). Most likely the observed contractile and metabolic transitions are not primary triggering events that render an aged skeletal muscle more susceptible to the loss of tissue mass and contractile strength, but a pathophysiological consequence of age-related abnormalities (54). It is important to stress that histological studies have established that certain aged human skeletal muscles exhibit a considerable reduction in fibre numbers, a drastic decline in fibre size and high levels of atrophying fast-twitching type II contractile fibres (21,27,29). Thus, the overall fast-to-slow transformation and glycolytic-to-oxidative metabolic shift during human skeletal muscle aging, as revealed by proteomics, possibly reflects the preferential loss of fast vs. slower contracting fibres in the senescent organism, and not an age-dependent adaptation due to enhanced muscle plasticity.

In agreement with the concept of age-related fibre type shifting, as exemplified by increased levels of slow contractile proteins and mitochondrial marker enzymes, is the observation of a higher concentration of the muscle-specific CA3 isoform (55) in aged human *vastus lateralis* muscle. This relatively abundant enzyme has not been previously identified by proteomic surveys of human skeletal muscle aging (38,52). CAs are widely distributed throughout the body and catalyse the reversible hydration of CO_2_ (66). The various isoforms play a crucial role in the acid-base balance, CO_2_-removal and provision of CO_2_ for metabolic processes, such as gluconeogenesis and the urea cycle, as well as the regulatory processes of ion homeostasis (67–69). Mammalian skeletal muscles express several CA isoforms in a fiber type-specific manner, whereby the predominant CA3 isoform is mostly present in the cytosolic fraction of type I and IIa fibers (55). Changes in neuromuscular activity patterns, metabolic alterations, stretch-induced hypertrophy and disuse atrophy have profound effects on the expression of muscle CAs (70–72). Of note, soluble isoforms of muscle CA are routinely used in clinical applications for assessing fibre damage (73). It has been suggested that the muscle-specific CA3 isoform presents a more sensitive biomarker of muscle damage compared to creatine kinase in neuromuscular disorders (74). Thus, CA is a well-established biomarker routinely used in muscle pathology (75), making it also an ideal candidate for evaluating potential fibre type shifting during muscle aging. The CA3 isoform levels were shown to be increased in senescent human skeletal muscle by both proteomics and independent immunoblot analysis. Although a higher concentration of CA3 may be due to an increased demand for efficient CO_2_-removal during fibre aging, the altered density of this abundant fibre type marker is more likely indicative of age-related fibre type shifting to slower-contracting muscle populations. In contrast to elevated levels of CA3 in aging human skeletal muscle, proteomic studies have recently established that the same muscle-specific CA isoform is decreased in dystrophic skeletal muscle (76) and non-obese, diabetic skeletal muscle (77). This suggests that the age-dependent increase in CA3 levels may be relatively specific with respect to sarcopenia in old age.

Other altered muscle-associated proteins of interest were Annexin A5, DJ-1 protein, glycogen phosphorylase, Hsp β-7 and Hsp70, creatine kinase, calmodulin and phosphoglucomutase. Increased levels of creatine kinase indicate a compensatory effect on the creatine phosphate shuttle system in aged muscle (78). An altered concentration of Annexin V and calmodulin may be due to altered ion handling during aging (79). Since calmodulin is a critical factor for Ca^2+^-sequestration and Ca^2+^-cycling during excitation-contraction coupling (80) and also plays a critical role in skeletal muscle plasticity (81), a changed density of this abundant Ca^2+^-binding protein presents an excellent new marker of age-related alterations in ion homeostasis. Since many metabolic enzymes involved in glycolysis, gluconeogenesis and glycogen metabolism are multi-functional (82), the alterations in phosphoglucomutase and glycogen phosphorylase during muscle aging are difficult to interpret. Both enzymes are of crucial importance for the bioenergetic utilization of muscle glucose. Phosphoglucomutase catalyses the inter-conversion of glucose-1-phosphate and glucose 6-phosphate, and glycogen phosphorylase facilitates the phosphorolytic cleavage of a glucosyl-residue from the glycogen polymer (83). The decreased levels of glycogen phosphorylase and increased density of phosphoglucomutase indicate altered flux rates of glucose and glycogen metabolism in senescent fibres. It has previously been shown that proteins involved in the cellular stress response are changed in various neuromuscular disorders (84), including sarcopenia in old age (40). The expression levels of various molecular chaperones have been shown to be altered after the fifth decade of life. However, distinct differences appear to exist between rodent models of aging and senescent human muscle (43). Of note, the mostly uncharacterised DJ-1 protein, which has been identified in this study by MS analysis, has previously been shown to play a role in neuro-degeneration (85). This makes this muscle-associated protein a potential new marker of sarcopenia.

In conclusion, aging of the human *vastus lateralis* muscle is associated with a plethora of proteome-wide changes, especially affecting fibre contraction, ion homeostasis, muscle metabolism and the cellular stress response. These proteomic findings are in agreement with findings from previous histological, physiological and biochemical investigations that have established cycles of denervation and impaired re-innervation causing the loss of entire motor units and muscular atrophy, excitation-contraction uncoupling at the triad junction, altered functioning of the actomyosin apparatus resulting in weaker contractility, impaired regulation of bioenergetic processes, disturbed ion handling and an altered cellular stress response by molecular chaperones. Importantly, the proteomic data presented in this study, are in agreement with altered levels of key metabolic enzymes and contractile elements that suggest a fast-to-slow transition of the contractile apparatus and a glycolytic-to-oxidative shift in metabolism during human muscle aging (54). The establishment of a novel biomarker signature of muscle aging after the fifth decade of life might be exploitable for the future design of a more reliable assay to diagnose sarcopenia of old age.

## Figures and Tables

**Table I.** List of DIGE-identified proteins with a changed abundance in middle aged vs. aged human vastus lateralis muscle.

Spot no.	Name of identified protein	Accession no.	Molecular mass (kDa)	Iso-electric point p*I*	Mascot score	Coverage (%)	Peptides matched	Anova	Fold change	Sequences
1	Actin, α skeletal muscle	gi|33563240	42372	5.23	135	28	10	2.32E-04	−3.8	AGFAGDDAPR, AVFPSIVGRPR, HQGVMVGMGQK, DSYVGDEAQSK, DSYVGDEAQSKR, LDLAGR, DLTDYLMK, GYSFVTTAER, LCYVALDFENEMATAASSSSLEK, SYELPDGQVITIGNER
2	Actin, α skeletal muscle	gi|33563240	42372	5.23	435	36	11	3.05E-04	−2.6	AGFAGDDAPR, DSYVGDEAQSK, DSYVGDEAQSKR, VAPEEHPTLLTEAPLNPK, DLTDYLMK, GYSFVTTAER, LCYVALDFENEMATAASSSSLEK, SYELPDGQVITIGNER, DLYANNVMSGGTTMYPGIADR, EITALAPSTMK, IIAPPER
3	Annexin A5	gi|6753060	35788	4.83	46	5	2	0.002	−2.5	VLTEIIASR, MLVVLLQANR
4	DJ-1 protein	gi|55741460	20240	6.32	47	36	6	7.00E-03	−2.1	ALVILAK, GAEEMETVIPVDVMR, VTVAGLAGK, GLIAAICAGPTALLAHEVGFGCK, DGLILTSR, APLVLKD
5	Troponin T, slow skeletal muscle	gi|3449358	29992	6.34	198	19	4	1.00E-03	−2	VDFDDIHR, DLLELQTLIDVHFEQR, VLSNMGAHFGGYLVK, YEINVLYNR
6	Troponin T, slow skeletal muscle	gi|3449358	29992	6.34	223	22	6	4.00E-03	−2	IPEGERVDFDDIHR, VDFDDIHR, DLLELQTLIDVHFEQR, KVLSNMGAHFGGYLVK, VLSNMGAHFGGYLVK, YEINVLYNR
7	Glycogen phosphorylase	gi|6755256	97689	6.65	60	8	8	6.00E-03	−1.9	VIFLENYR, VIPAADLSEQISTAGTEASGTGNMK, GYNAQEYYDR, GYNAQEYYDRIPELR, IPELR, DIVNMLMHHDR, TIAQYAR, EIWGVEPSR
8	Heat shock protein β-7	gi|31542970	18660	5.95	44	19	3	1.20E-02	−1.8	ALPAQDPPMEK, LAADGTVMNTFAHK, EDGSLTIR
9	Actin, β	gi|49868	39451	5.78	101	23	10	2.00E-03	−1.7	AVFPSIVGR, HQGVMVGMGQK, DSYVGDEAQSK, DSYVGDEAQSKR, LDLAGR, DLTDYLMK, SYELPDGQVITIGNER, EITALAPSTMK, IIAPPER, IIAPPERK
10	Troponin C, slow	gi|6678369	18525	4.04	121	24	3	5.00E-03	−1.7	AAVEQLTEEQKNEFK, GKSEEELSDLFR, NADGYIDLDELK
11	Myosin light chain MLC2	gi|199985	18870	4.71	100	40	6	6.00E-03	−1.5	DTFAALGR, EAPGPINFTVFLTMFGEK, GADPEETILNAFK, VFDPEGK, EMLTTQAER, NLVHIITHGEEKD
12	Acyl-CoA dehydrogenase short chain	gi|192659	45208	8.96	153	12	4	1.30E-02	1.3	GISAFLVPMPTPGLTLGK, IAMQTLDMGR, LADMALALESAR, ITEIYEGTSEIQR
13	Heat shock-protein Hsp70	gi|31560686	69889	5.51	175	12	7	6.00E-03	1.4	VEIIANDQGNR, TTPSYVAFTDTER, EIAEAYLGGK, DAGTITGLNVLR, IINEPTAAAIAYGLDK, FEELNADLFR, VCNPIISK
14	Carbonic anhydrase 3	gi|31982861	29638	6.89	47	8	2	1.20E-02	1.5	VVFDDTYDR, YAAELHLVHWNPK
15	Creatine kinase M-type	gi|6671762	43250	6.58	86	12	4	1.00E-02	1.6	GGDDLDPNYVLSSR, LSVEALNSLTGEFK, IEEIFK, GQSIDDMIPAQK
16	Succinate dehydrogenase complex subunit A	gi|3851614	59266	6.16	77	4	2	1.30E-02	1.6	LGANSLLDLVVFGR, SMQNHAAVFR
17	Actin, α-cardiac	gi|387090	42048	5.23	358	21	9	3.00E-03	1.7	AGFAGDDAPR, HQGVMVGMGQK, DSYVGDEAQSK, DSYVGDEAQSKR, DLTDYLMK, GYSFVTTAER, SYELPDGQVITIGNER, CDIDIRK, IIAPPER
18	Calmodulin	gi|99032084	16696	4.09	59	47	6	5.00E-03	1.7	EAFSLFDKDGDGTITTK, MKDTDSEEEIR, DTDSEEEIREAFR, VFDKDGNGYISAAELR,DGNGYISAAELR, EADIDGDGQVNYEEFVQMMTAK
19	Phosphoglucomutase	gi|33416468	63705	6.02	69	9	5	4.35E-05	2	VDLGVLGK, SMPTSGALDR, FFGNLMDASK, YDYEEVEAEGANK, LSGTGSAGATIR

## Abbreviations:

2D: 2-dimensional;
CA: carbonic anhydrase;
DIGE: difference in-gel electrophoresis;
Hsp: heat shock protein;
IEF: isoelectric focusing;
MS: mass spectrometry;
SDS: sodium dodecyl sulfate;
PAGE: polyacrylamide gel electrophoresis;
PBS: phosphate-buffered saline
